# Orange‐Fleshed Sweet Potatoes, Grain Amaranth, Biofortified Beans, and Maize Composite Flour Formulation Optimization and Product Characterization

**DOI:** 10.1002/fsn3.70455

**Published:** 2025-06-18

**Authors:** Julius Byamukama, Robert Mugabi, Dorothy Nakimbugwe, John Muyonga

**Affiliations:** ^1^ Department of Food Technology and Nutrition, School of Food Technology, Nutrition and Bioengineering, CAES Makerere University Kampala Uganda

**Keywords:** biofortified crops, composite flours, extrusion, micronutrients, response surface methodology

## Abstract

Blending different ingredients can enhance the nutritional value of foods to address the needs of vulnerable populations. This study aimed to develop optimal formulations for raw and extruded flours from orange‐fleshed sweet potatoes (OFSP), grain amaranth, biofortified beans, and maize. Blends were extruded using a twin‐screw extruder at a barrel temperature of 60°C, 120°C, and 150°C, a screw speed of 350 rpm, and 5% feed moisture. The formulations were optimized using a response surface methodology, with the aim of identifying optimal formulations for raw (RF) and extruded (EF) flours, without (1) or with (2) maize. Formulation optimization was based on the following ranges: OFSP (20%–68%), maize (0%–30%), biofortified beans (20%–40%), and grain amaranth (10%–30%). The response variables were protein, beta‐carotene, iron, zinc, and viscosity. Quadratic and cubic models accurately predicted the variables (*p* < 0.05) with *R* ^2^ values between 0.93 and 1.00. The first (RF1) and second (RF2) optimal formulations for raw composite flours were OFSP (37.8%), biofortified beans (32.2%), and grain amaranth (30.0%), and OFSP (20.0%), maize (30.0%), biofortified beans (40.0%), and grain amaranth (10.0%), respectively. For extruded composite flours, the optimal formulations (EF1) and (EF2) were OFSP (46.6%), biofortified beans (36.9%), and grain amaranth (16.5%), and OFSP (49.0%), maize (5.9%), biofortified beans (35.1%), and grain amaranth (10.0%), respectively. Formulation and treatment had significant effects on flour composition, pasting properties, and phytochemical content. Extrusion lowered viscosity, while formulations without maize had higher phytochemical content.

## Graphical abstract

Optimal formulations for raw and extruded flours from orange‐fleshed sweet potatoes (OFSP), grain amaranth, biofortified beans, and maize were developed. Optimized formulations contained higher proteins, fibre and phytochemicals than a commercial cereal‐soybean flour. Formulation and treatment had significant effects on flour composition, functional properties and phytochemical content. Extrusion lowered viscosity, while formulations without maize had higher phytochemical content.

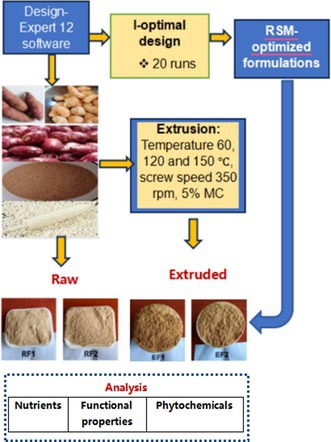

## 1 Introduction

Composite flours are blends of flours made from a variety of ingredients, which typically include tubers, roots, grains, and legumes. Production of composite flours is regarded as a strategy for enhancing the nutritional profile, lowering the cost of food products, or improving functionality (Marchini et al. 2021). Composite flours can be used in a variety of food products, including porridges, pasta, noodles, and bakery products to achieve desired nutritional and functional outcomes (Chiedu et al. 2023; Masri et al. 2014; Natocho et al. 2024). However, ensuring a balanced nutritional profile during the formulation of composite flours can be challenging. Additionally, some flours, such as beans, grain amaranth and soybeans, may introduce anti‐nutritional factors such as phytates, trypsin inhibitors, oxalates and tannins (Nafula et al. 2022; Zinia et al. 2022). Anti‐nutrients interfere with digestion and nutrient absorption in the body, resulting in low nutrient bioavailability (Nafula et al. 2022). Processing techniques such as extrusion cooking have been proposed as ways of reducing anti‐nutrients and enhancing nutritional quality, as well as functional and sensory properties of flours (Awolu et al. 2017; Nkundabombi et al. 2016).

Porridges are widely used as weaning or breakfast food in Uganda (Malik et al. 2015; Nansereko et al. 2022), but they are usually inadequate in terms of protein, essential amino acids, vitamin A, and minerals content (Ndagire et al. 2015; Tibagonzeka 2014). This is because porridges offered to young children in Uganda are mainly made from one type of flour, such as maize flour, millet flour, sorghum flour, cassava flour, or rice flour, which makes them nutritionally unbalanced (Akande et al. 2017). Several research efforts have been made towards improving the quality of flours used in making porridges for young children by blending cereal crops with other food materials to form composite flours such as millet with skimmed milk powder and vegetables (Tumwine et al. 2019), amaranth leaves with skimmed milk and OFSP (Tumuhimbise et al. 2019), millet with pigeon pea or red gram (Chakraborty et al. 2011), and maize and soybean protein concentrate (Omosebi et al. 2018).

Orange‐fleshed sweet potato (OFSP) ( *Ipomoea batatas* L.) is a vital biofortified crop rich in beta‐carotene and a good source of energy, vitamins, and minerals (Mwanga et al. 2016; Neela and Fanta 2019). Biofortified beans and grain amaranth are important sources of iron, zinc, complex carbohydrates, dietary fiber, and antioxidants (Mulambu et al. 2017; Saltzman et al. 2013; Tibagonzeka 2014). Incorporation of OFSP, biofortified beans, and grain amaranth in flour for porridges has been reported as an efficient strategy to improve nutritional value and reduce micronutrient deficiencies among children under the age of 5 years (Aderibigbe et al. 2022; Stangoulis and Knez 2022).

Extrusion cooking is a high‐temperature, short‐time process that minimizes anti‐nutritional factors that inhibit protein functionality and digestibility (Gu et al. 2017). It is a versatile and effective method of transforming raw protein‐rich or starchy materials into finished food products (Julianti et al. 2017). The unique heat processing of this technology has been utilized to produce a wide range of products such as ready‐to‐eat expanded products formulated with carrot pomace (Alam et al. 2015), blends of OFSP, sorghum, and soybean (Alawode et al. 2017), precooked grain amaranth flour (Atukuri et al. 2019), composite flour from sweet potato, maize, soybean, and xanthan gum (Julianti et al. 2017) and extruded corn‐based snacks (Diaz et al. 2013). Despite the benefits of extrusion cooking, most flours consumed in Uganda are not extruded. There is hence a need to have nutrient‐enhanced formulations for raw flours to cater for the large population segment that do not have access to extruded flours.

Response surface methodology (RSM) is a valuable statistical technique used for multiple regression analysis with measurable variables (Yolmeh and Jafari 2017). The technique can be used in food product development to optimize and improve many elements of food products, ultimately improving performance and process efficiency (Malik et al. 2015) and optimizing the formulation and processing conditions to produce desired sensory and nutritional qualities, while minimizing production costs (Natocho et al. 2024). Response surface methodology has been used to research a variety of products, such as functional noodles (Natocho et al. 2024), dried jackfruit pulp (Nansereko et al. 2022), supplementary bean‐based composite flour (Ndagire et al. 2015), among others. However, information on optimizing the formulation of composite flour containing OFSP, biofortified bean, and grain amaranth is limited. This study therefore investigated the use of response surface methodology in the production of nutrient‐enhanced raw and precooked OFSP‐based composite flour, with grain amaranth and biofortified beans as the other universal ingredients and maize flour as an optional ingredient. Maize was considered due to its abundance, while the other ingredients were included due to their high nutritional value.

## 2 Materials and Methods

### 2.1 Raw Materials

Grain amaranth (*A. hypochondriacus L*.) was purchased from a local industry located in Mukono district. Biofortified beans (NARObean 2) and orange‐fleshed sweet potatoes (NAPSPOT12 0) were purchased from the National Crops Resources Research Institute (NACRRI) Namulonge, Wakiso district. The commercial composite flour (control) used in the study was purchased from a retail supermarket in Kampala, Uganda, and was made of maize, soybean, and rice. The reagents used were of analytical grade (AR), manufactured by GRIFFCHEM Fine Chemicals (India) and Loba Chemie PVT Ltd. (India).

### 2.2 Preparation of Grain Amaranth Flour

Grain amaranth flour was prepared following the method of Atukuri et al. (2019). Grain amaranth was washed, sorted to eliminate extraneous matter, and dried in a hot air oven (Gallenkamp, Uk) at 55°C for 6 h. Clean grain amaranth was milled using a commercial mill (30B‐C, Changzhou Erbang Drying Equipment Co. Ltd) and sieved with a mesh size of 500 μm. The resulting flour was packaged in airtight bags and stored at room temperature (25°C ± 5°C) for subsequent use.

### 2.3 Preparation of Orange Fleshed Sweet Potato Flour

The OFSP roots were sorted, trimmed, washed manually with clean water, peeled, and sliced into uniform sizes of 2 mm thickness using an electrical machine (Ritter E16, Ritterwerk GmbH, Grobenzell, Germany). The slices were soaked in 5 g/L sodium metabisulfite solution for 5 min and excess water was drained from the samples after 5 min as described by (Chikpah et al. 2020) with slight modification. About 1000 g of the slices were spread out on perforated trays and dried at 60°C air temperature using a Hohenheim HT min, cabinet dryer (innotech‐ingenieursgesellschaft GmbH, Altdorf, Germany). The dried slices were milled into flour using a mill (Kenwood blender BLP10), and the flour was packaged in a polyethylene bag and stored at room temperature (25°C ± 5°C) for subsequent use.

### 2.4 Processing of Extruded Bean Flour

Extruded bean flour was produced using a twin‐screw extruder (model DP 70 III Jinan, China) with three heating sections: the first at 60°C, the second at 120°C, and the last one at 150°C. The screw speed was at 350 rpm, and the cutters at 90 rpm, the diameter was 4 mm, and the flour was extruded at 5% moisture content. The extrusion conditions were based on previous experiments on the optimization of extrusion conditions. Extrudates were ground to a fine flour using a Wonder mill (Pocatello, Idaho, USA) and sieved through a 425 μm mesh size. The flour was packaged in a polyethylene bag and stored at room temperature (25°C ± 5°C) for subsequent use.

### 2.5 Experimental Design for Optimizing the Formulation of OFSP‐Based Composite Flour

I‐optimal design of Response Surface Methodology (Design Expert Stat‐Ease, version 12.0.10.0 32‐bit, Minneapolis, USA) was used. The input variables were OFSP (20%–68%), maize flour (0%–30%), biofortified bean flour (20%–40%), and grain amaranth (10%–30%) which generated 20 experimental runs. The response variables were protein content, beta carotene content, iron content, zinc content, and viscosity and they were expressed individually as a function of the input variables. The upper and lower limits for independent variables (Table 1) were decided based on the preliminary and literature studies. The four best formulations obtained from the optimization result were used for further analyses. Regression analysis was used to determine the relationship between response variables (*Y*); protein content, beta carotene content, iron content, zinc content, and viscosity.

**TABLE 1:** Coded and actual levels of mixture components used to generate experimental runs.

Component	Symbol	Codes and corresponding levels		
		−1	0	+1
OFSP	*X*_1_	20	44	68
Maize	*X*_2_	0	15	30
Beans	*X*_3_	20	30	40
Grain amaranth	*X*_4_	10	20	30

The equation below was used:

$$Y=\beta 0+\beta_{1}X_{1}+\beta_{2}X_{2}+\beta_{11}X_{12}+\beta_{22}X_{22}+\beta_{12}X_{1}X_{2}$$

Where *β*_0_, *β*_1_, *β*_2_, *β*_11_, *β*_22_, and *β*_12_ represent regression coefficients for interception, linear, quadratic, and interactive effects, respectively, *X*_1_, *X*_2_, *X*_3_, and *X*_4_ are actual independent variables, and *Y* is the response variable.

### 2.6 Preparation of Optimized OFSP‐Based Composite Flours

Response surface methodology was used to determine the optimum formulation of nutrient‐enhanced OFSP‐based composite flours. The desirability function approach (DFA) was used to simultaneously optimize the composite flours' protein content, beta carotene content, iron content, zinc content, and viscosity in both raw and extruded composite flours. The desirability function approach method incorporates desires and priorities for each of the variables, and the maximum desirability is 1. Four optimal formulations were selected from optimization results. These included RF1 (raw, without maize), RF2 (Raw with maize), EF1 (extruded without maize) and EF2 (extruded with mainze). The details of optimal combinations used to prepare OFSP‐based composite flours are indicated in Table 2.

**TABLE 2:** Optimal formulations used in the production of OFSP‐based composite flours.

Sample ID	OFSP (%)	Grain amaranth (%)	Biofortified bean (%)	Maize flour (%)
RF1	37.8	30.0	32.2	0.0
RF2	20.0	10.0	40.0	30.0
EF1	46.6	16.5	36.9	0.0
EF2	49.0	10.0	35.1	5.9

Abbreviations: EF1, EF2: extruded composite formulations; RF1, RF2: raw composite formulations.

### 2.7 Validation of Optimized Formulations

Nutrient enhanced OFSP‐based composite flours were produced using the optimized levels of incorporation of OFSP, biofortified bean, grain amaranth, and maize as determined by design Expert software, and experimental values of the response variables were determined and compared with the theoretical values using *t*‐test (*p* < 0.05) in Microsoft Excel 2021 to establish the validity of predictions.

### 2.8 Analytical Methods

#### 2.8.1 Carotenoids Determination

Analysis of beta carotene was done using the HarvestPlus method (Tiony and Irene 2021).

#### 2.8.2 Determination of Moisture Content

Moisture content was determined using the Air Oven Method, AOAC method no. 925.10 (AOAC 2016).

#### 2.8.3 Crude Fat

The fat in samples was determined using the Soxhlet method, AOAC method no. 922.10 (AOAC 2016).

#### 2.8.4 Ash Content

Ash content was determined using AOAC method no. 923.03 (AOAC 2016).

#### 2.8.5 Dietary Fiber

Dietary fiber of OFSP‐based composite flour was determined using AOAC method no. 2011.25 (AOAC 2016).

#### 2.8.6 Crude Protein

Protein content was determined based on the Kjeldahl method, AOAC Method no. 920.87 (AOAC 2016), using a Kjeltec 8200 Auto Distillation Unit, model: 10014901.

#### 2.8.7 Carbohydrates

Carbohydrates content was determined by the difference method (Huber and BeMiller 2024).

$$\begin{array}{c} \text{Carbohydrates}\left(\%\right)\;100-\\ \left(\%\text{Moisture}+\%\text{Crude protein}+\%\mathrm{Fat}+\%\mathrm{Ash}\right) \end{array}$$

#### 2.8.8 Determination of Gross Energy

The total energy of OFSP‐based composite flours was determined based on the amounts of protein, fat, and carbohydrate present in the sample using Atwater factors (Sanchez‐Pena et al. 2017).

#### 2.8.9 Zinc and Iron

The amount of zinc and iron in the composite flour was measured by Atomic Absorption Spectroscopy (PerkinElmer, AAnalyst 700, USA), according to AOAC (2010). Results were expressed as mg/100 g sample (Tiony and Irene 2021).

#### 2.8.10 Bulk Density

Bulk density was determined according to the method described by (Chandra et al. 2015).

#### 2.8.11 Water Absorption Capacity (WAC) and Oil Absorption Capacity (OAC)

Water absorption capacity and oil absorption capacity were determined by the method described by Chandla et al. (2017).

#### 2.8.12 Water Absorption and Solubility Index

The water absorption and solubility were determined using the method described by Atukuri et al. (2019). The water absorption index and water solubility were calculated using the following equations.

$$\mathrm{WAI}=\frac{\text{Weight of sediment}\;\left(g\right)}{\text{Weight of the sample}\;\left(g\right)}$$

$$\mathrm{WSI}\;\left(\%\right)=\frac{\text{Weight of dissolved solid in spuernatant}\;\left(g\right)}{\text{Weight of the sample}\;\left(g\right)}\times 100$$

#### 2.8.13 Swelling Capacity

The method described by Chandla et al. (2017) was used to determine the swelling power of the sample.

#### 2.8.14 Pasting Properties

Pasting characteristics were determined using a Rapid Visco Analyzer (Perten Instruments AB, Kungens Kurva, Sweden) as described by RVA method no. 01.05. Pasting parameters namely: peak viscosity, trough, breakdown, final viscosity, setback, peak time, and pasting temperatures were read from the pasting profile.

#### 2.8.15 Total Phenolic Content

The total phenolic content was determined by Folin–Ciocalteu assay according to Hussain et al. 2022, with some modifications. Briefly, 500 μL was mixed with 500 μL Folin–Ciocalteu solution, then 1 mL sodium bicarbonate (10% solution) was added to the mixture and finally adjusted to the mark with distilled water to make a 10 mL solution. Then, the mixture was vortexed for 60 s. The solutions were left for 35 min at room temperature in a dark place. Afterwards, the absorbance was measured at 765 nm using a spectrophotometer (Spectroquant pharo 300, Darmstadt, EU).

#### 2.8.16 Total Flavonoid Content

Total flavonoid content was measured using the colorimetric method described by Doloking et al. (2022), with some modifications. Briefly, 1 mL of extracts was mixed with 4 mL of distilled water and 0.3 mL of 5% sodium nitrite solution in 15 mL falcon tubes. The tubes were allowed to stand for 5 min, and 0.3 mL of 10% aluminium chloride was added to the mixture and allowed to stand for 1 min. Lastly, 2 mL of 1 M sodium hydroxide and 2.4 mL of distilled water were added and mixed thoroughly. After centrifugation for 5 min at 2253× *g*, the tubes were kept in a dark place at room temperature for 40 min. Absorbance was read at 510 nm using a spectrophotometer (Spectroquant pharo 300, Darmstadt, EU) against a blank prepared similarly by replacing the mixture with methanol. The total flavonoid content was calculated from a standard curve of quercetin, and the results were expressed as mg of quercetin equivalent per gram of dry sample (mg QE/g DM).

#### 2.8.17 Determination of Total Antioxidants

The total antioxidant activity of composite flour was determined using a free radical scavenging assay by the use of 1,1‐diphenyl‐2‐picrylhydrazyl (DPPH) as the source of the free radicals (Hussain et al. 2022).

#### 2.8.18 Determination of Total Condensed Tannins

Total condensed tannins were determined using the method described by Hussain et al. (2022) with slight modifications. Briefly, 1.5 mL of vanillin solution (4%) *w*/*v* was added to 50 μL of sample extract in a test tube. Immediately after, 0.75 mL of concentrated HCl was added and the mixture was vortexed for 60 s. The mixture was incubated at room temperature for 10 min to allow for color development. Absorbance was read at 500 nm using a spectrophotometer (Spectroquant pharo 300, Darmstadt, EU) with water as a blank. A standard curve was developed using catechin standards of varying concentrations (0.02 to 0.06 mg/mL). Total condensed tannins values were expressed as mg catechin equivalent/100 g of the sample.

#### 2.8.19 Phytates

Phytates were determined using a spectrophotometer (spectroquant pharo 300, Darmstadt, EU) as described by Nwosu et al. (2014).

### 2.9 Statistical Analysis

Design Expert Software (Stat‐Ease, version 12.0.10.0 32‐bit, Minneapolis, USA) was used for optimization of input variables to obtain optimal response variables (beta‐carotene, protein, iron, zinc, and viscosity). The significance of the models was determined using Analysis of Variance (ANOVA). Models' lack‐of‐fit and coefficient of determination (*R* ^2^) tests were used to test the adequacy of the developed models. The study used analysis of variance (ANOVA) and Fisher Least Significant Difference (LSD) test using XLSTAT software version 2018.1 (Addinsoft, New York, NY, USA) to compare the properties of the different composite flours, with significance accepted at *p* < 0.05. All composition data were reported on a dry matter basis. Experimental results were expressed as the means ± standard deviations (SD).

## 3 Results and Discussion

### 3.1 Effect of Extrusion Conditions on the Nutritional Quality of OFSP‐Based Composite Flour

#### 3.1.1 Protein Content

**FIGURE 1:**
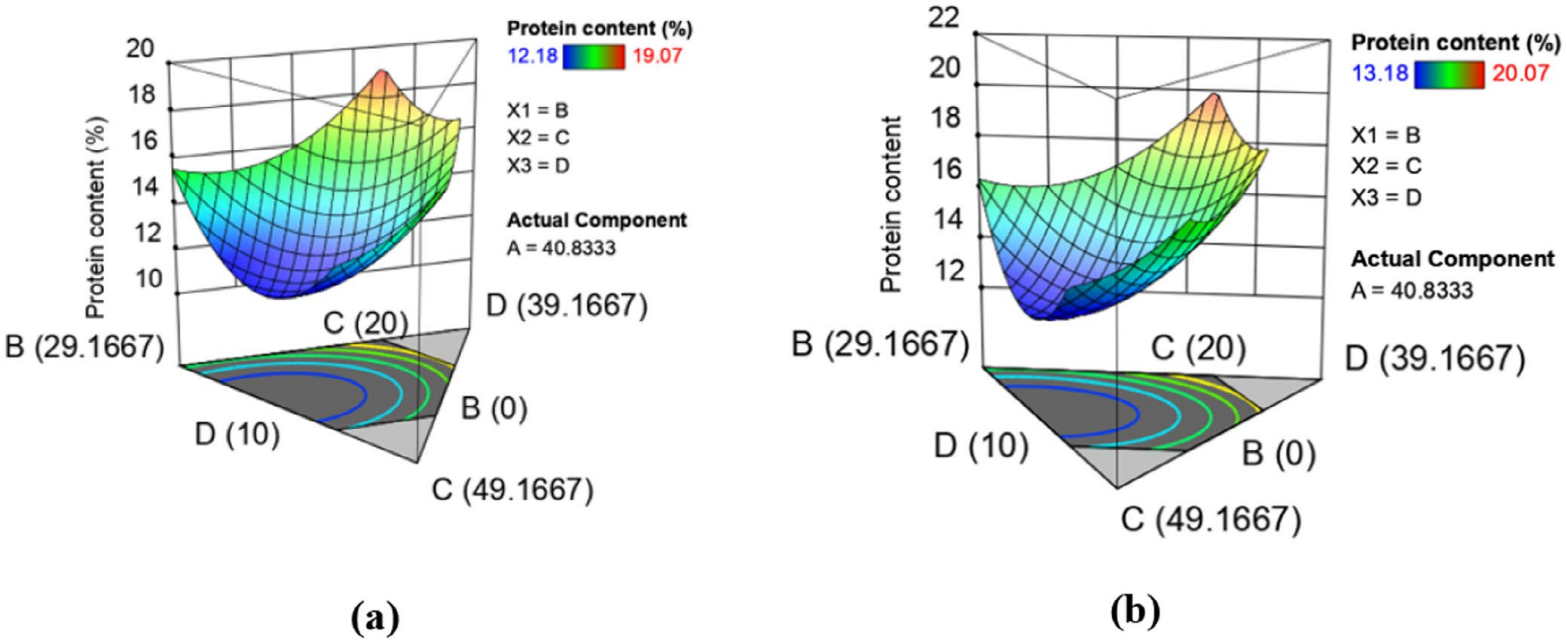
Response surface plots showing effects of varying levels of orange‐fleshed sweet potatoes, maize, biofortified beans, and grain amaranth flours on protein content in raw (a) and extruded (b) composite flours.

In this study, the protein content ranged from 13.18% to 20.07% in raw and 12.18% to 19.07% in extruded (Table 3) composite flours. Extrusion cooking caused a decrease in the protein content of the extruded composite flours compared to their raw counterparts. Similar trends were reported in other extrusion studies (Akande et al. 2017; Nicole et al. 2010), in studies to optimize the extrusion conditions for the production of instant grain amaranth‐based porridge flour and characterization of ready‐to‐eat composite porridge flours made by soy‐maize‐sorghum‐wheat extrusion cooking process. According to Omosebi et al. (2018), a decrease in the protein content of extruded products could be attributed to protein denaturation and apparent partial loss of certain amino acids along with other nitrogenous compounds on heating. Mosibo et al. (2022) noted that high temperatures favor Maillard reactions leading to the degradation of amino acids such as lysine which is easily degraded because of its two available reactive amino groups. The effect of formulation on the protein content was determined and is represented by equations (1) and (2) which show the models in terms of actual levels of variables in raw and extruded composite flours, respectively. Figure 1 shows the response surface plots for the effect of interaction of input variables in raw (a) and extruded (b) on protein content. The fitted models for protein content in raw and extruded composite flour runs are given below;

(1) $$\begin{array}{c} \text{Protein content}=-0.039808X_{1}-0.695767X_{2}+0.226570X_{3}\\ +1.62788X_{4}+0.036530X_{1}X_{2}+0.008981X_{1}X_{3}\\ -0.013466X_{1}X_{4}+0.036460X_{2}X_{3}-0.018743X_{2}X_{4}\\ -0.017876X_{3}X_{4}-0.001605X_{1}X_{2}X_{3}+0.00018X_{1}X_{2}X_{4}\\ -0.000412X_{1}X_{3}X_{4}-0.000263X_{2}X_{3}X_{4}\\ \left(p=<0.0001,R^{2}=9.9999,\text{lack of}\;\mathrm{fit}=0.7558\right) \end{array}$$

(2) $$\begin{array}{c} \text{Protein content}=-0.026786X_{1}-0.657536X_{2}+0.218763X_{3}\\ +1.54239X_{4}+0.034857X_{1}X_{2}+0.0088835X_{1}X_{3}-0.012145X_{1}X_{4}\\ +0.035171X_{2}X_{3}-0.016891X_{2}X_{4}-0.01582X_{3}X_{4}-0.00153X_{1}X_{2}X_{3}\\ +0.000170X_{1}X_{2}X_{4}-0.000412X_{1}X_{3}X_{4}-0.000278X_{2}X_{3}X_{4}\\ \left(p<0.000,R^{2}=0.9895,\text{lack of}\;\mathrm{fit}=0.7029\right) \end{array}$$

where *X*_1_, *X*_2_, *X*_3_, and *X*_4_ are actual values of OFSP, maize, biofortified beans, and grain amaranth flours respectively.

**TABLE 3:** I‐optimal design arrangement and responses for raw and extruded OFSP‐based composite flours.

Input variables					Response variables									
Run	Sweet potato (%)	Maize (%)	Beans (%)	Grain amaranth (%)	Raw					Extruded				
					Protein (%)	Beta carotene (mg/100 g)	Iron (mg/100 g)	Zinc (mg/100 g)	Viscosity (cP)	Protein (%)	Beta carotene (mg/100 g)	Iron (mg/100 g)	Zinc (mg/100 g)	Viscosity (cP)
1	20	23.8	36.1	20.0	16.02	0.35	1.95	0.69	899	15.76	0.19	2.95	1.65	75
2	30.3	11.1	28.5	30.0	18.38	0.55	2.05	0.74	827	17.46	0.32	3.05	0.92	70
3	20.0	23.8	36.1	20.0	16.08	0.37	1.99	0.62	825	14.42	0.16	2.92	1.64	77
4	68.0	1.3	20.5	10.0	13.43	2.12	0.7	0.19	703	12.54	1.76	1.7	0.67	67
5	55.9	7.2	20.0	16.7	14.68	1.87	0.85	0.18	827	13.42	1.65	1.85	0.65	87
6	46.8	11.9	31.2	10.0	13.20	1.51	1.45	0.19	678	12.24	1.34	2.45	1.21	85
7	32.1	23.8	20.7	23.2	16.91	0.44	1.62	0.28	908	15.95	0.37	1.62	0.69	85
8	20.0	30.0	20.0	30.0	18.71	0.31	1.83	0.69	1321	17.84	0.25	2.83	0.65	87
9	50.9	0.0	31.9	17.1	15.69	1.83	1.81	0.36	505	14.78	1.54	2.81	1.26	63
10	38.8	3.5	40.0	17.6	15.58	0.51	2.02	0.69	533	14.25	0.34	3.02	1.73	72
11	33.5	30.0	26.5	10.0	13.18	0.45	0.82	0.28	1321	12.28	0.48	1.82	0.85	86
12	50.9	0.0	31.9	17.1	15.64	1.86	1.86	0.66	492	14.75	0.61	2.82	1.27	65
13	34.9	15.0	40.0	10.0	13.35	0.55	2.25	1.1	648	12.92	0.48	3.25	1.72	70
14	30.3	11.1	28.5	30.0	18.35	0.54	2.06	0.77	820	17.53	0.31	3.06	0.84	70
15	33.5	30.0	26.5	10.0	13.21	0.46	0.82	0.15	1113	12.34	0.49	1.82	0.86	86
16	30.0	0.0	40.0	30.0	20.07	0.62	2.28	1.23	525	19.75	0.36	3.28	1.73	63
17	40.8	0.0	30.7	28.4	17.97	1.31	2.12	0.95	497	16.63	0.9	3.12	1.16	64
18	44.5	16.3	20.5	18.5	15.96	1.50	0.92	0.17	862	14.76	1.21	1.92	0.67	72
19	50.0	0.0	20.0	30.0	18.07	1.73	0.67	0.13	494	17.86	1.31	1.67	0.65	66
20	44.5	16.3	20.5	18.5	15.99	1.56	0.96	0.11	862	14.96	1.24	1.97	0.69	73

#### 3.1.2 Beta Carotene

**FIGURE 2:**
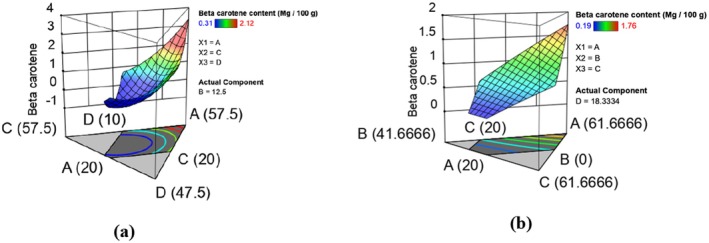
Response surface plots showing effects of varying levels of orange‐fleshed sweet potatoes, maize, biofortified bean, and grain amaranth flours on beta carotene content in raw (a) and extruded (b) composite flours.

The beta carotene content from experimental runs was in the range 0.31–2.65 mg/100 g in raw and 0.19–1.76 mg/100 g for extruded composite flour (Table 3). Extrusion cooking caused a significant decrease in beta‐carotene content. Similar trends were observed by Akande et al. (2017). The reduction in beta carotene content could be attributed to thermal degradation, which appears to be the major factor contributing to beta carotene loss during extrusion (Waramboi et al. 2013). The effect of formulation on beta carotene was determined and is represented by equations (3) and (4) which show the models in terms of actual levels of variables. Figure 2 shows the response surface plots for the effect of formulation in raw (a) and extruded (b) on beta‐carotene content. The fitted models for beta carotene in raw and extruded composite flour runs are given below:

(3) $$\begin{array}{c} \beta =0.078647X_{1}-0.565532X_{2}-0.3014X_{3}-0.072724X_{4}\\ +0.015213X_{1}X_{2}+0.009528X_{1}X_{3}+0.005394X_{1}X_{4}+0.022892X_{2}X_{3}\\ +0.011414X_{2}X_{4}+0.009687X_{3}X_{4}+0.000517X_{1}X_{2}X_{3}+0.000176X_{1}X_{2}X_{4}\\ +0.0003X_{1}X_{3}X_{4}+0.000331X_{2}X_{3}X_{4}\\ \left(p=<0.0001,R^{2}=0.9905,\text{lack of}\;\mathrm{fit}=0.9565\right) \end{array}$$

(4) $$\begin{array}{c} \beta =0.042124X_{1}-0.011253X_{2}+0.001409X_{3}-0.008159X_{4}\\ +0.000123X_{1}X_{2}+0.000607X_{1}X_{3}\;\\ \left(p=<0.0001,R^{2}=0.8376,\text{lack of}\;\mathrm{fit}=0.7632\right) \end{array}$$

where *X*_1_, *X*_2_, *X*_3_ and *X*_4_ are actual values of OFSP, maize, biofortified bean and grain amaranth flours respectively.

#### 3.1.3 Iron

**FIGURE 3:**
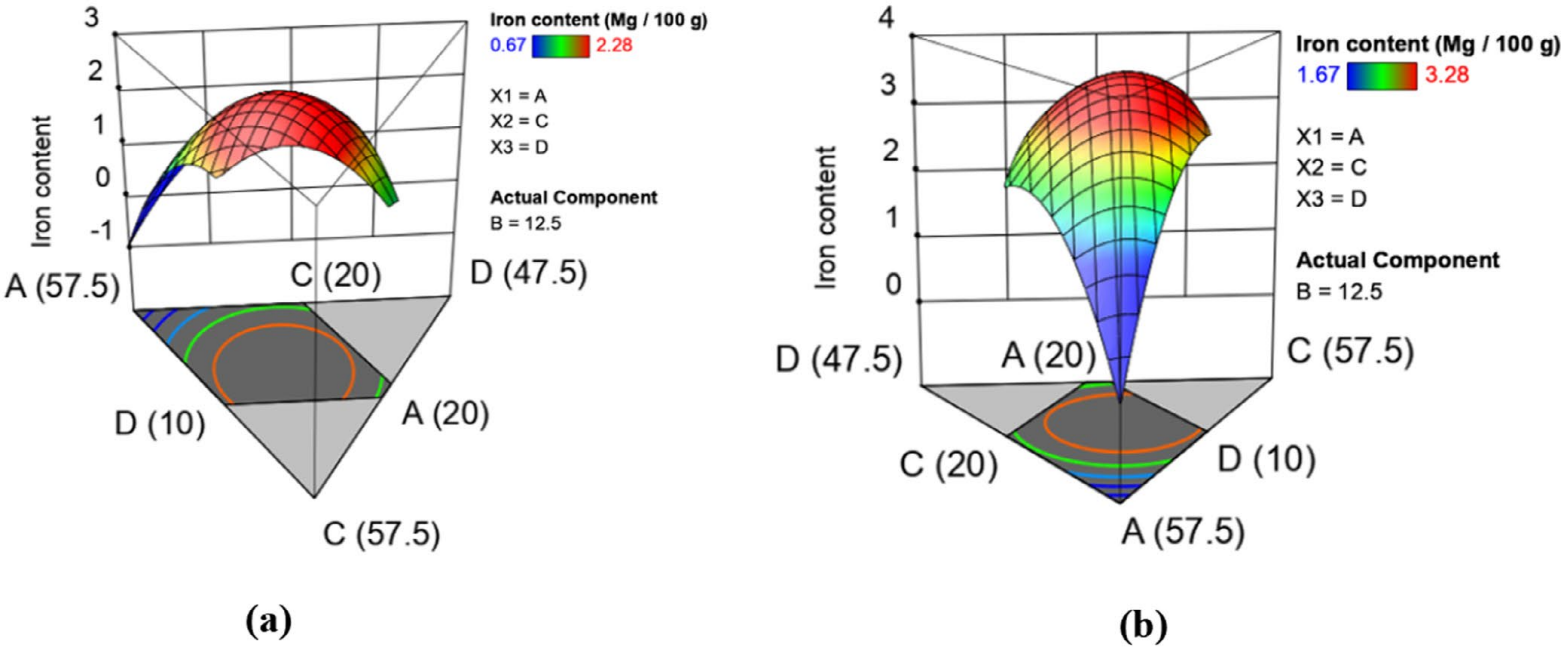
Response surface plots showing effects of varying levels of orange‐fleshed sweet potatoes, maize, biofortified bean, and grain amaranth flours on iron content in raw (a) and extruded (b) composite flours.

The iron content in raw and extruded composite flour runs was in the range of 0.67–2.28 and 1.67–3.28 mg/100 g, respectively (Table 3). Extrusion cooking increased the iron content of the extruded composite flour runs. This is in agreement with Nicole et al. (2010), in a study on the characterization of ready‐to‐eat composite porridge flours made by the soy‐maize‐sorghum‐wheat extrusion cooking process. Omwamba and Mahungu (2014) also noted an increase in iron content due to extrusion in a study that aimed to develop a protein‐rich ready‐to‐eat extruded snack from a composite blend of rice, sorghum, and soybean flour. According to Akande et al. (2017), an increase in iron content during extrusion cooking is usually attributed to an improvement in its bioavailability by reducing other factors such as phytates that can bind iron and form insoluble iron‐phytate complexes that reduce the availability of iron in the sample. This binding makes it difficult for the iron to be detected (Sun et al. 2021). Bioavailability measures the extent and rate at which the active ingredient or active moiety is absorbed and becomes available at the site of action, and a poorly bioavailable nutrient may not be present in detectable concentrations (Skov et al. 2019). A previous study revealed that the presence of phytates in legumes and grains can significantly decrease the availability of iron, leading to lower AAS results when measuring iron content (Akomo et al. 2016). The effect of formulation on iron content was determined and is represented by equations (5) and (6) which show the models in terms of actual levels of variables. Figure 3 shows the response surface plots for the effect of formulation in raw (a) and extruded (b) on iron content. The fitted models for iron content in raw and extruded composite flour runs are given below:

(5) $$\begin{array}{c} \mathrm{Fe}=0.004921X_{1}-0.054957X_{2}-0.225802X_{3}-0.4806X_{4}\\ -0.012851X_{1}X_{2}+0.002067X_{1}X_{3}\\ +0.00439X_{1}X_{4}+0.006816X_{2}X_{3}+0.017109X_{2}X_{4}\\ +0.01313X_{3}X_{4}+0.000189X_{1}X_{2}X_{3}+0.000189X_{1}X_{2}X_{4}\\ +0.000094X_{1}X_{3}X_{4}-0.000556X_{2}X_{3}X_{4}\\ \left(p=<0.000,R^{2}=0.9998,\text{lack of}\;\mathrm{fit}=0.8569\right) \end{array}$$

(6) $$\begin{array}{c} \mathrm{Fe}=0.01491X_{1}-0.044941X_{2}-0.215813X_{3}-0.470663X_{4}\\ -0.012852X_{1}X_{2}+0.002067X_{1}X_{3}+0.004391X_{1}X_{4}\\ +0.006815X_{2}X_{3}+0.017110X_{2}X_{4}+0.013134X_{3}X_{4}\\ +0.000389X_{1}X_{2}X_{3}+0.000189X_{1}X_{2}X_{4}+0.000094X_{1}X_{3}X_{4}\\ -0.000556X_{2}X_{3}X_{4}\\ \left(p=<0.0001,R^{2}=1.0000,\text{lack of}\;\mathrm{fit}=0.8471\right) \end{array}$$

where *X*_1_, *X*_2_, *X*_3_, and *X*_4_ are actual values of OFSP, maize, biofortified bean and grain amaranth flours respectively.

#### 3.1.4 Zinc

**FIGURE 4:**
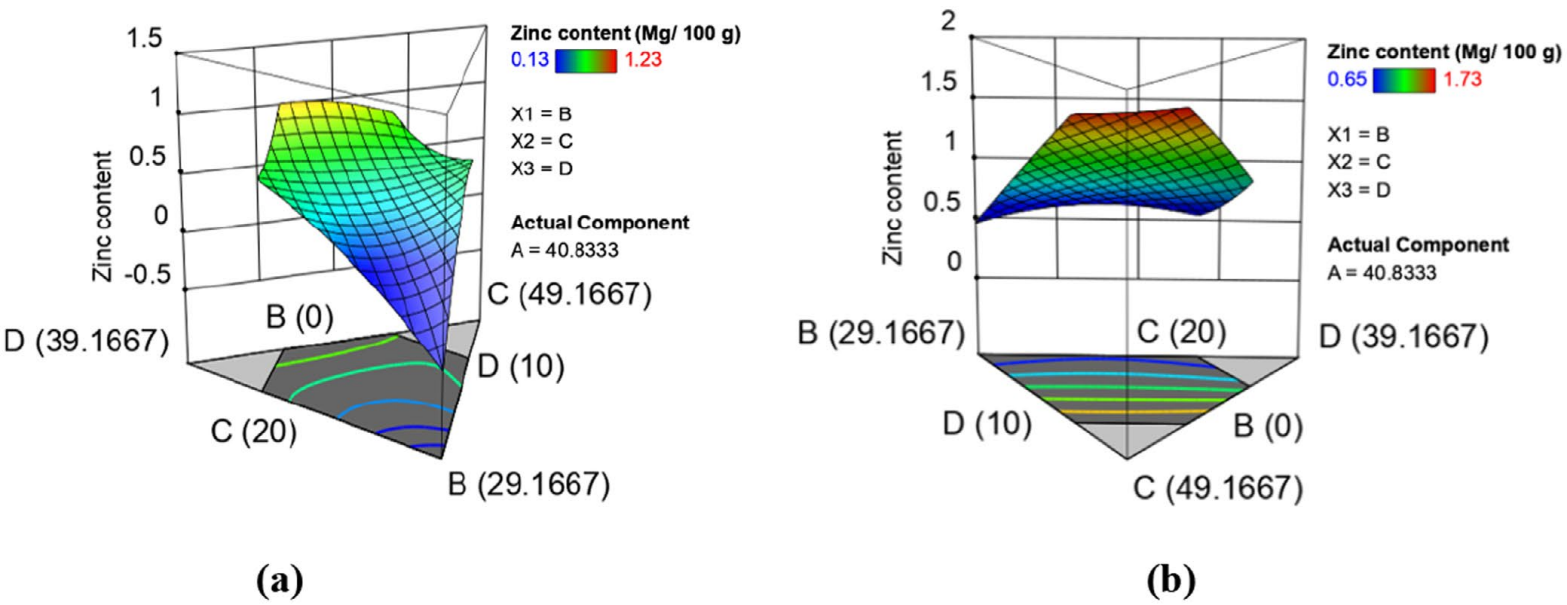
Response surface plots showing effects of varying levels of orange‐fleshed sweet potatoes, maize, biofortified bean, and grain amaranth flours on zinc in raw (a) and extruded (b) composite flours.

Zinc content within the runs of raw and extruded composite flours was in the range of 0.13–1.23 mg/100 g and 0.15–1.23 mg/100 g, respectively (Table 3). Extrusion cooking increased the zinc content of the extruded composite flour runs. Similar increases were reported by Akande et al. (2017), in a study to optimize the extrusion conditions for the production of instant grain amaranth‐based porridge flour. An increase in zinc content can be attributed to the release of phytate to release phosphate molecules, partly attributed to the destruction of polyphenols and reorganization of dietary fiber components changing their chelating properties by extrusion cooking (Nahemiah et al. 2015). The effect of formulation on zinc content was determined and is represented by equations (7) and (8) which show the models in terms of actual levels of variables. Figure 4 shows the response surface plots for the effect of formulation in raw (a) and extruded (b) on zinc content. The fitted models for zinc content in raw and extruded composite flour runs are given below:

(7) $$\begin{array}{c} \mathrm{Zn}=0.2456X_{1}-1.53X_{2}+1.31X_{3}+3.97X_{4}\\ +1.42X_{1}X_{2}-2.29X_{1}X_{3}-6.62X_{1}X_{4}+13.36X_{2}X_{3}\\ +0.0278X_{2}X_{4}-7.23X_{3}X_{4}-14.76X_{1}X_{2}X_{3}\\ +7.15X_{1}X_{2}X_{4}+29.07X_{1}X_{3}X_{4}-44.70X_{2}X_{3}X_{4}\\ \left(p=0.0027,R^{2}=0.9642,\text{lack of}\;\mathrm{fit}=0.4293\right) \end{array}$$

(8) $$\begin{array}{c} \mathrm{Zn}=0.002729X_{1}+0.01127X_{2}+0.097462X_{3}\\ -0.063501X_{4}-0.000376X_{1}X_{2}-0.001094X_{1}X_{3}\\ -0.001026X_{1}X_{4}-0.000950X_{2}X_{3}+0.000896X_{2}X_{4}\\ \left(p=<0.0001,R^{2}=0.9886,\text{lack of}\;\mathrm{fit}=0.126\right) \end{array}$$

where *X*_1_, *X*_2_, *X*_3_, and *X*_4_ are actual values of OFSP, maize, biofortified bean, and grain amaranth flours, respectively.

#### 3.1.5 Viscosity

**FIGURE 5:**
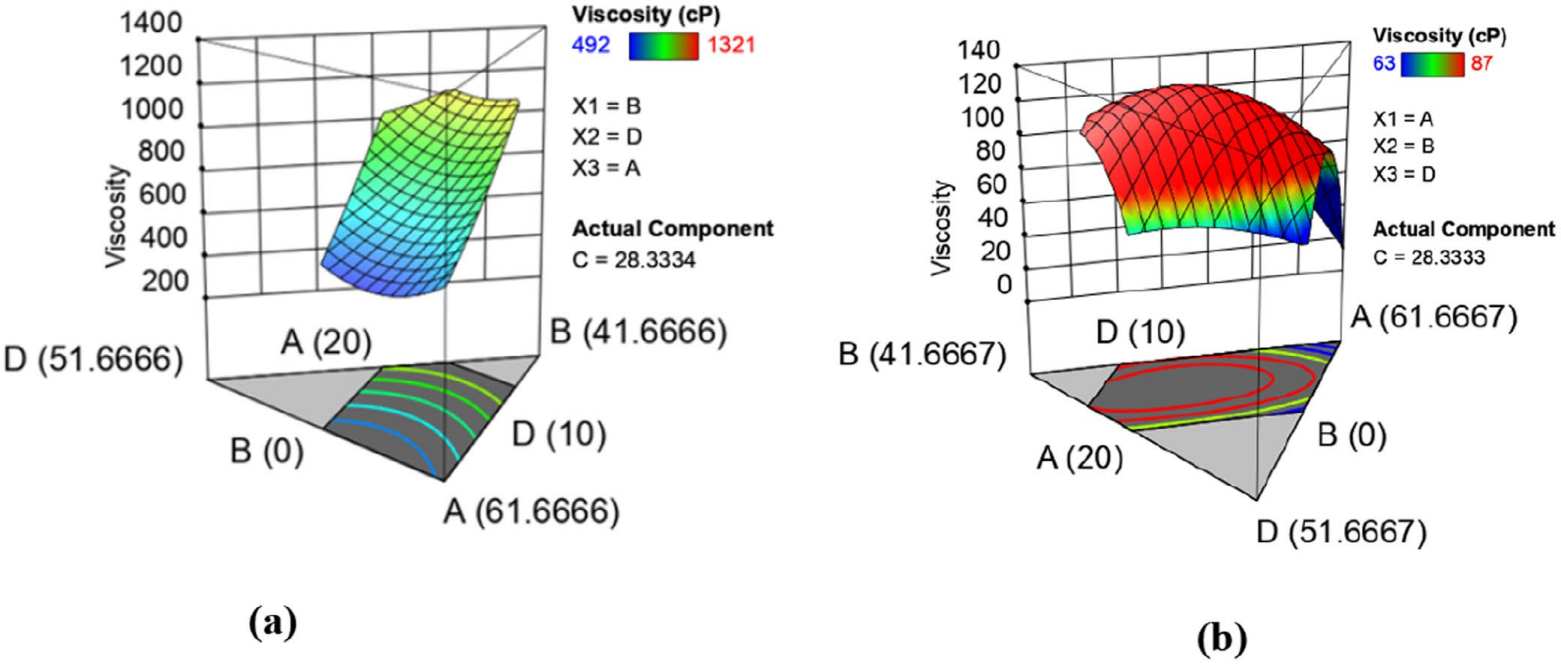
Response surface plots showing effects of varying levels of orange‐fleshed sweet potatoes, maize, biofortified bean, and grain amaranth flours on viscosity in raw (a) and extruded (b) composite flours.

Viscosity is important in food intake because it contributes to an increase or decrease in the bulk of a cooked cereal product and affects taste intensity. The viscosity of gruels made from the flour samples varied between 492 and 1321 cp and 63 and 87 cp (Table 3) for the runs of raw and extruded composite flours, respectively. Similar trends were reported by Akande et al. (2017) in a study to optimize the extrusion conditions for the production of instant grain amaranth‐based porridge flour. Low viscosity is desirable in the preparation of infants and weaning foods (Nicole et al. 2010). The lower viscosities (higher flour rate) of the extruded composite flour runs could be attributed to higher starch damage during extrusion cooking. Extrusion cooking causes swelling and rupture of the granules (Byaruhanga et al. 2014), modification of crystalline spectra (Gu et al. 2017), an increase in cold water solubility, and a reduction in viscosity of the starch and release of amylose and amylopectin (Julianti et al. 2017). The effect of formulation on viscosity was determined and is represented by equations (9) and (10) which show the models in terms of actual levels of variables. Figure 5 shows the response surface plots for the effect of formulation in raw (a) and extruded (b) on viscosity. The fitted models for viscosity in raw and extruded composite flour runs are given below:

(9) $$\begin{array}{c} V_{s}=13.80599X_{1}+46.31747X_{2}-911.62263X_{3}\\ +39.37868X_{4}-0.168037X_{1}X_{2}+0.129428X_{1}X_{3}\\ -0.844362X_{1}X_{4}-0.266651X_{2}X_{3}-0.702302X_{2}X_{4}\\ +0.027511X_{3}X_{4}\\ \;\left(p=<0.0001,R^{2}=0.9348,\text{lack of}\;\mathrm{fit}=0.3581\right) \end{array}$$

(10) $$\begin{array}{c} V_{s}=1.0534X_{1}+22.45529X_{2}-8.42708X_{3}-45.6922X_{4}\\ -0.90059X_{1}X_{2}-0.009818X_{1}X_{3}+0.697866X_{1}X_{4}\\ -0.893241X_{2}X_{3}+0.695356X_{2}X_{4}+1.04902X_{3}X_{4}\\ +0.039158X_{1}X_{2}X_{3}-0.004818X_{1}X_{2}X_{4}-0.003783X_{1}X_{3}X_{4}\\ \;\left(p=<0.0001,R^{2}=0.9970,\text{lack of}\;\mathrm{fit}=0.1442\right) \end{array}$$

where *X*_1_, *X*_2_, *X*_3_, and *X*_4_ are actual values of OFSP, maize, biofortified bean, and grain amaranth flours, respectively.

### 3.2 Optimal Formulation of OFSP‐Based Composite Flours

During optimization, the importance of the response variable (beta carotene content, protein content, iron content, zinc content, and viscosity) was set at 3 for all. The selected optimum levels of the different input variables (OFSP, biofortified bean, grain amaranth, and maize) with predicted values for the response variables (beta carotene, protein, iron, zinc, and viscosity) generated by the software are shown in Table 4. The desirability function approach (DFA) was used to simultaneously optimize the composite flours' protein content, beta carotene content, iron content, zinc content, and viscosity in raw and extruded OFSP‐based composite flours. The desirability function approach method incorporates desires and priorities for each of the variables, with 0 representing a completely undesirable value of response, whereas 1 represents the maximum desirability.

**TABLE 4:** Optimal solutions in raw and extruded composite flours.

Solutions	Sweet potato (%)	Maize (%)	Beans (%)	Grain amaranth (%)	Protein content (%)	Beta carotene content (mg/100 g)	Iron content (mg/100 g)	Zinc content (mg/100 g)	Viscosity (cP)	Desirability	
Raw composite flours											
1	**37.81**	**0.00**	**32.19**	**30.00**	**17.97**	**1.27**	**2.12**	**1.16**	**555.56**	**0.80**	**Selected**
2	38.69	0.00	31.31	30.00	17.88	1.35	2.06	1.12	554.18	0.77	
3	39.45	0.00	30.55	30.00	17.79	1.41	2.01	1.09	552.84	0.76	
4	**20.00**	**30.00**	**40.00**	**10.00**	**17.17**	**1.36**	**2.55**	**2.81**	**908.71**	**0.74**	**Selected**
Extruded composite flours											
1	**46.60**	**0.00**	**36.87**	**16.53**	**16.79**	**0.88**	**2.57**	**1.23**	**34.69**	**0.76**	**Selected**
2	39.81	0.00	40.00	20.18	17.11	0.59	2.92	1.92	52.21	0.75	
3	34.68	0.00	35.64	29.68	19.21	0.47	3.28	0.73	57.27	0.71	
4	**49.01**	**5.88**	**35.11**	**10.00**	**15.28**	**1.01**	**2.17**	**1.35**	**37.56**	**0.70**	**Selected**

*Note:* The values in bold indicate the optimal formulations in raw and extruded composite flours selected by design expert software.

Two combinations with the highest desirability value from raw composite flours (0.8) and (0.7) were selected which corresponded to formulations consisting of 37.8% OFSP, 0.0% maize, 32.2% bean, 30% grain amaranth, and 20% OFSP, 30% maize, 40% bean, and 10% grain amaranth respectively. The predicted values for response variables for composite flours formulated using these optimal formulations were 17.97% protein content, 1.27 mg beta carotene, 2.12 mg iron, 1.16 mg zinc, 555.56 cP viscosity and 17.17% protein, 1.36% beta carotene, 2.55 mg iron, 2.81 mg zinc, and 908.7 cP viscosity as indicated in Table 4.

Two combinations with the highest desirability value from extruded composite flours (0.8) and (0.7) were also selected, which corresponded to formulations consisting of 46.6% OFSP, 0.0% maize, 36.9% bean, 16.5% grain amaranth and 49.0% OFSP, 5.9% maize, 35.1% bean, and 10.0% grain amaranth, respectively. The predicted values for response variables for composite flours formulated using these optimal formulations were 16.79% protein content, 0.88% beta carotene, 2.57 mg iron, 1.23 mg zinc, 34.7 cP viscosity and 15.28% protein, 1.01 mg beta carotene, 2.17 mg iron, 1.35 mg zinc, and 37.56 cP viscosity, as shown in Table 4.

### 3.3 Functional Properties of Raw and Extruded OFSP‐Based Composite Flours

Functional properties are intrinsic physicochemical characteristics and are usually linked to the interaction of food components with water and oil (Kraithong et al. 2018). They include: water absorption capacity, oil absorption capacity, water absorption index, water solubility index, swelling power, and many others. Functional properties can be used to foresee the technological impact of a given ingredient on a food product (Pardhi et al. 2019). The changes in hydration properties during extrusion can be attributed to structural changes in polymeric (starch and protein) and non‐polymeric (lipids) macromolecular components (Syed et al. 2021). These properties are influenced mainly by the size and structure of starch granules (Sahu and Patel 2021). The results of functional properties of the control and OFSP‐based composite flours are presented in Table 5.

**TABLE 5:** Functional and pasting properties of OFSP‐based composite flours.

Sample ID	Functional properties						Pasting properties						
	Bulk density (g/mL)	WAC (%)	OAC (%)	WAI (g/g)	WSI (%)	Swelling power (%)	Peak viscosity (RVU)	Trough (RVU)	Break down (RVU)	Final viscosity (RVU)	Setback (RVU)	Peak time (min)	Pasting temperature (°C)
COMM	0.776^a^ ± 0.009	227.713^d^ ± 1.899	187.642^e^ ± 1.602	5.231^a^ ± 0.101	2.365^d^ ± 0.243	7.879^a^ ± 0.511	2449^a^ ± 7.94	1362.33^a^ ± 5.86	1083.33^a^ ± 7.37	4056^a^ ± 5.29	2686^a^ ± 5.57	5.73^c^ ± 0.01	81.13 ± 1.02
RF1	0.592^b^ ± 0.005	230.056^cd^ ± 2.423	241.096^d^ ± 5.153	5.40^a^ ± 0.070	15.100^c^ ± 0.674	10.740^b^ ± 0.640	362.00^c^ ± 4.00	229.67^b^ ± 9.24	338.33^c^ ± 11.93	405.33^b^ ± 4.04	209.33^c^ ± 12.50	6.69^b^ ± 0.03	N/D
RF2	0.606^b^ ± 0.005	239.449^c^ ± 3.327	251.813^c^ ± 3.593	4.861^b^ ± 0.001	14.996^c^ ± 0.481	8.550^b^ ± 0.314	432.33^b^ ± 8.14	119.33^c^ ± 11.59	415.33^b^ ± 7.02	387.67^c^ ± 8.62	261.33^b^ ± 0.58	6.93^a^ ± 0.13	N/D
EF1	0.527^c^ ± 0.008	528.571^a^ ± 1.796	275.018^b^ ± 0.435	2.576^c^ ± 0.145	50.401^b^ ± 2.450	6.921^c^ ± 0.149	125.33^e^ ± 7.77	22.00^d^ ± 1.00	6.67^d^ ± 0.58	37.00^d^ ± 2.00	15.33^d^ ± 0.57	1.52^d^ ± 0.07	N/D
EF2	0.515^c^ ± 0.002	499.710^b^ ± 2.284	295.126^a^ ± 1.954	2.153^d^ ± 0.110	57.810^a^ ± 1.920	6.904^c^ ± 0.207	216.33^d^ ± 4.51	17.00 ^cd^ ± 1.00	15.00^d^ ± 2.00	33.00^d^ ± 0.00	16.67^d^ ± 0.58	1.19^e^ ± 0.12	N/D

*Note:* Results are presented as mean ± SD of samples. Means in the same column with the same superscript were not significantly (*p* > 0.05) different. Values with different superscripts in the same columns are significantly different, whereas values with the same superscript in the same columns are not significantly different.

Abbreviations: COMM: commercial composite flour (control); EF1 and EF2: extruded composite formulations; OAC: oil absorption capacity; RF1 and RF2: raw composite formulations; WAC: water absorption capacity; WAI: water absorption index; WSI: water solubility index.

#### 3.3.1 Bulk Density (BD)

The bulk density measures the heaviness of a flour product (Nicole et al. 2010). The bulk density of samples ranged between 0.515 and 0.776 g/mL. The highest BD was recorded in the control (COMM) and the least BD in the extruded formulation EF2. The low bulk density values in extruded formulations could be attributed to the effect of heating (Kesselly et al. 2023). Extrusion cooking has a reducing effect on the BD of flour because of starch hydrolysis to simple sugars such as maltose and dextrin that alter the structure and particle size of the flour (Gui et al. 2012). Additionally, a decrease in bulk density in extruded formulations could also be due to starch gelatinization (Wang et al. 2015). Gelatinization increases the volume of extruded products which results in a decrease in bulk density (Alam et al. 2015). Similar findings have been observed by (Kolawole et al. 2018).

#### 3.3.2 Water Absorption Capacity (WAC)

Water absorption capacity is the ability of the flour to absorb or associate with water and swell for improved consistency and texture in food (Dereje et al. 2020). The ability of a food material to absorb water largely depends on its protein and starch contents (Byaruhanga et al. 2014). The WAC ranged between 227.713% and 528.571% for all flours evaluated in this study. The highest WAC was observed in the extruded formulation (EF1) and the lowest WAC in the control (COMM). Kolawole et al. (2018) also reported higher WAC for extruded flour compared to raw ones. The high WAC recorded in extruded formulations could be due to starch depolymerization forming small and uniform particle sizes that have a high surface area and capillarity (Kesselly et al. 2023). Water absorption capacity is a desirable parameter in food systems to improve yield and consistency under limited water conditions such as in dough and paste (Eke‐Ejiofor et al. 2021). It also gives body to the food (Chandra et al. 2015).

#### 3.3.3 Oil Absorption Capacity (OAC)

Oil absorption capacity measures the ability of a product to entrap oil (Jeyakumari et al. 2016). It is an important functional parameter to consider in food processing because the oil acts as a flavor retainer and improves the mouth feel of foods, improves palatability, and extends the shelf life of foods, especially bakery products (Julianti et al. 2017). The oil absorption capacity ranged between 187.642% and 295.126% in all flours. The highest OAC was observed in EF2 and the lowest in the control. Extrusion cooking caused a significant (*p* < 0.05) increase in OAC. Similar observations have been reported in earlier studies (Filli et al. 2013; Ito et al. 2015). The high OAC in extruded OFSP‐based composite flours could be linked to a high level of starch degradation as a result of exposure to high thermal energy (Patil and Kaur 2018). The high OAC of the composite flours indicates that the flours could also be used in making bakery products. According to Kesselly et al. (2023) ingredients with a high OAC allow the stabilization of high fat food products and emulsions.

#### 3.3.4 Water Absorption Index (WAI)

The water absorption index is a measure of the volume the starch occupies after swelling in excess water and is an indication of the integrity of starch (Dereje et al. 2020). It measures the water absorbed by starch and can be used as an indicator for gelatinization, an important property of starches. The WAI for flours evaluated in this study was between 5.40 and 2.153 g/g in all flours, with the highest recorded in RF1 and the lowest in EF2. The higher values of WAI in RF1 formulation could be attributed to the high protein content present due to the high proportion of grain amaranth (Gelaye 2023). Proteins have numerous hydrophilic sites that bind water (Kesselly et al. 2023). The low values of WAI in extruded formulations (EF1 and EF2) could be attributed to starch gelatinization caused by extrusion temperature (Gu et al. 2017). Gelatinized starch has been found to absorb a limited amount of water (Castro et al. 2020). The study findings are in agreement with those of Atukuri et al. (2019) and Alam et al. (2015), who reported a decrease in water absorption index in extruded grain amaranth flour and a cereal‐based ready‐to‐eat expanded product formulated with carrot pomace. In complementary foods, a high water absorption index is undesirable because it contributes to dietary bulk (Mosibo et al. 2022). During cooking, foods with high WAI absorb large amounts of water to form voluminous low‐energy and nutrient foods (Awolu et al. 2017). Therefore, the low water absorption values observed for extruded flours in this study are desirable as they are appropriate for making thinner gruels which have high caloric density per unit volume (Alam et al. 2015).

#### 3.3.5 Water Solubility Index (WSI)

The water solubility index represents the solubility of biomolecules including starches, water‐soluble fiber, proteins, and sugar in water (Atukuri et al. 2019; Otondi et al. 2020). The WSI of flours was least for the control (2.37%) and highest for the extruded formulation EF2 (57.81%). Eke‐Ejiofor et al. (2021) also reported lower WSI for raw than extruded complementary flour made from OFSP starch supplemented with soybean and ground nut flour. The high WSI in extruded formulations can be attributed to dextrinization, gelatinization, and depolymerization of starch forming low molecular weight compounds (amylose and amylopectin) (Awolu 2017). Furthermore, it has been noted by several researchers that the water solubility index increases with a decrease in feed moisture (Chakraborty et al. 2011; Filli et al. 2013; Ito et al. 2015).

#### 3.3.6 Swelling Power

Swelling power is the ability of starch to absorb water and cause granules to increase in size (Awolu 2017). It is indicative of the degree of water absorption of the starch granules in the flour (Julianti et al. 2017). The swelling power was found to be highest for RF1 (10.74%) and the least for EF2 (6.904%). Swelling power was affected by extrusion but not by formulation. The swelling power of flour depends on the size of flour particles, source, and type of processing (Chandra et al. 2015). The low value of swelling power recorded in the extruded sample could be attributed to a reduction in the number of starch granules due to disruption and gelatinization during extrusion (Otondi et al. 2020). Gelatinized starch imbibes limited amounts of water and exhibits limited swelling (Nkesiga 2023). The low swelling power observed in extruded flours makes them suitable for use in the preparation of gruels used as weaning foods, as they will result in porridges of low viscosity desirable for children (Atukuri et al. 2019).

### 3.4 Pasting Properties of Raw and Extruded OFSP‐Based Composite Flours

The results of pasting properties of the control and OFSP‐based composite flours are presented in Table 5. Peak viscosity is the maximum viscosity acquired by the starch granule before physical rupture (Kesselly et al. 2023), and an indicator of the thickening behavior and water holding capacity of starch (Chikpah et al. 2020). High peak viscosity indicates high starch content. The control had the highest peak viscosity (2449 RVU), whereas the extruded formulation EF1 had the least (125.33 RVU). The low peak viscosities observed in extruded formulations might be attributed to starch modification due to high temperature, pressure, and shear forces involved during extrusion (Byaruhanga et al. 2014). The low peak viscosity exhibited by OFSP‐based composite flours is suitable for products requiring low gel strength and elasticity. This property makes composite flour a suitable weaning food for children because they require porridge with low viscosity (Atukuri et al. 2019). Kesselly et al. (2023) found that peak viscosities of extruded cowpeas flour were greatly reduced compared with corresponding unextruded flours, an observation attributable to extrusion causing partial gelatinization of starch.

Breakdown viscosity determines the stability of starch when heated under constant shear (Ocheme et al. 2017). Breakdown viscosity ranged from 6.67 to 1083.33 RVU. Generally, extruded samples exhibited low breakdown viscosities. The control reported the highest breakdown and was significantly (*p* < 0.05) different from the rest of the samples. A decrease in breakdown viscosities were reported in earlier extrusion studies (Akinwale et al. 2017; Awolu et al. 2017). Low breakdown viscosity exhibited by the extruded samples is an indication of their ability to withstand breakdown during heating and shearing (Ocheme et al. 2017).

Final viscosity is a measure of the ability of flour to form a viscous gel or paste during heating and cooling, and it is commonly used to define the quality of a particular starch‐based flour (Kesselly et al. 2023). It shows the change in the viscosity after holding cooked starch at 50°C. The final viscosity ranged from 33.0 to 4056 RVU, with extruded formulation EF2 having the lowest value, followed by extruded formulation EF1 (37.0 RVU), whereas the control had the highest final viscosity. The low final viscosities exhibited in OFSP‐based composite flours are nutritionally beneficial in the preparation of flours to be used in gruels for feeding individuals with high nutrient content since more flour can be incorporated in a given volume of gruel.

Setback is the viscosity after cooling to 50°C. The extent of increase in viscosity on cooling to 50°C reflects the retrogradation tendency, a situation that causes the paste to become firmer and increasingly resistant to enzyme attack, leading to reduced paste digestibility (Patil and Kaur 2018). Low setback indicates lower tendency for retrogradation and subsequently higher digestibility (Awolu et al. 2017). The extruded formulations had the lowest setback while the control had the highest setback. This could have been attributed to starch pre‐gelatinization caused by high temperature during extrusion (Ndagire et al. 2015). The low setback viscosities (15.33 and 16.67 RVU) for the extruded OFSP‐based composite flours indicates that their paste would have a high stability against retrogradation and suitable in the preparation of infant foods (Chikpah et al. 2020). It also implies that the porridge, when consumed by children, would be easy to digest (Nkundabombi et al. 2016).

The peak time is a measure of cooking time whereas the pasting temperature is the minimum temperature required by the starch to start gelatinizing (Julianti et al. 2017). The peak time ranged between 1.19 and 6.93 min whereas the pasting temperature was only detected in the control (81.1°C). These results can be attributed to the raw material composition and processing techniques employed (Bashir and Aggarwal 2019), as extrusion results in disruption of starch granules, loss of granule integrity, and crystallinity (Kesselly et al. 2023). The lower peak time obtained from OFSP‐based composite samples suggests a shorter cooking time and a lower energy consumption during cooking as compared to the control (Chikpah et al. 2020).

### 3.5 Proximate Analysis

The results for proximate composition of the control and OFSP‐based composite flours are summarized in Table 6.

**TABLE 6:** Proximate composition (%) of commercial (control), raw, and extruded OFSP‐based composite flours.

Sample ID	Moisture content	Crude protein	Dietary fiber	Ash	Crude fat	Carbohydrates	Gross energy (kcal)
COMM	7.72^b^ ± 0.15	15.45^c^ ± 0.02	0.46^e^ ± 0.08	1.39^c^ ± 0.03	5.65^a^ ± 0.21	69.79^ab^ ± 0.65	391.81^b^ ± 0.57
RF1	8.15^a^ ± 0.12	18.16^a^ ± 0.81	4.04^a^ ± 0.05	3.61^a^ ± 0.06	4.65^ab^ ± 0.07	65.43^c^ ± 1.43	376.21^a^ ± 1.56
RF2	7.84^ab^ ± 0.01	17.55^ab^ ± 0.50	3.39^b^ ± 0.16	2.95^b^ ± 0.07	2.35^c^ ± 0.21	69.31^b^ ± 0.41	368.59^e^ ± 0.7
EF1	5.84^d^ ± 0.00	16.39^bc^ ± 0.03	2.15^c^ ± 0.08	3.81^a^ ± 0.22	4.10^b^ ± 0.49	69.86^ab^ ± 0.54	381.90^c^ ± 1.52
EF2	6.33^c^ ± 0.19	16.13^c^ ± 0.43	1.45^d^ ± 0.19	3.16^b^ ± 0.19	2.30^c^ ± 0.28	72.08^a^ ± 2.65	373.54^ad^ ± 0.29

*Note:* Results are presented as means ± SD. Means in the same columns with different superscripts are significantly (*p* < 0.05) different. Values with different superscripts in the same columns are significantly different, whereas values with the same superscript in the same columns are not significantly different.

Abbreviations: COMM: commercial composite flour (control); EF1 and EF2: extruded composite formulations; RF1 and RF2: raw composite formulations.

The moisture content ranged between 5.84% and 8.15% in all samples. The extruded composite flour formulations had significantly (*p* < 0.05) lower moisture content compared to the raw ones. This could be attributed to the fact that during extrusion, the extrudates are released from a high pressure and temperature to a low pressure and temperature zone causing the product to expand and evaporate (Byaruhanga et al. 2014). A Similar range (4.79%–8.34%) for moisture content was reported by Honi et al. (2018) for extruded orange‐ fleshed sweet potato and bambara groundnut based snacks. The moisture content of all flour samples obtained in this study was within the acceptable limit for flours (not more than 10%) for the long storage (Alawode et al. 2017). Therefore, the moisture content of developed flours could confirm a longer shelf life (Tumuhimbise et al. 2019).

The crude protein content ranged from 15.45% to 18.16% for all formulations. The highest protein was observed in RF1, whereas the control had the least. Extrusion cooking caused a decrease in protein content, from 18.16% to 16.39% in formulations without maize and from 17.55% to 16.13% in formulations with maize. Similar trends were also reported by previous authors (Akande et al. 2017; Nicole et al. 2010) in a studies to optimize the extrusion conditions for the production of instant grain amaranth‐based porridge flour and characterization of ready‐to‐eat composite porridge flour made by soy‐maize‐sorghum‐wheat extrusion cooking process. According to Omosebi et al. (2018), a decrease in protein content in extruded flours could be attributed to protein denaturation and apparent partial loss of certain amino acids and nitrogenous compounds on heating. Mosibo et al. (2022) noted that high temperatures promote Maillard reactions, resulting in the degradation of amino acids.

The dietary fiber varied between 0.46% and 4.04%. The highest fiber content (4.04%) was obtained from the formulation without maize (RF1) and the least fiber (0.46%) was from commercial composite flour (control). The low fiber content in the formulation with maize could be attributed to the fact that processing of maize flour involves the removal of bran that mainly contains the fiber content of the maize (Kamotho 2020). The high value of crude fiber content in the RF1 formulation could be due to high levels of OFSP and biofortified beans incorporation because these materials have been reported to have high fiber content (Chikpah et al. 2020; Neela and Fanta 2019). The results obtained in this study were slightly lower than (5.29%–6.46%) reported in the study by Honi et al. (2018) on extruded orange‐fleshed sweet potato and bambara groundnut‐based snacks.

The highest ash content (3.81%) was obtained from extruded blend (EF1). The least ash content (1.39%) was observed in commercial composite flour (control). Formulations containing maize had significantly lower ash content compared to those without. This may be attributed to the fact that refined maize has low mineral content (Kamotho 2020). Minerals in the maize are mainly imbedded in the bran (Gallego‐Castillo et al. 2021), and the common processing method used for maize is huller milling, which involves the removal of bran and germ to make refined flour (Muyanja et al. 2020) which makes it inferior in terms of ash content. The observed results are in agreement with the study by Honi et al. (2018) who reported a range of 0.09%–4.80% ash content in extruded orange‐fleshed sweet potato and bambara groundnut based snacks. There was no significant (*p* < 0.05) difference in ash content between raw and extruded flours. This is probably because minerals are heat stable (Wani and Kumar 2016). Ash is an inorganic residue in any food substance that directly represents the mineral content (Tumwine et al. 2019).

The maximum (5.65%) and minimum (2.30%) crude fat contents were obtained from the commercial composite flour and the blend (EF2) respectively. Crude fat content in formulations containing maize (RF2 and EF2) was significantly lower compared to the crude fat content in formulations without maize (RF1 and EF1). This could be due to the refinement of maize flour that involves the removal of germ that mainly contains the fat (Gallego‐Castillo et al. 2021). The content of crude fat in extruded composite formulations (EF1 and EF2) was significantly (*p* < 0.05) lower than that in raw composite formulations (RF1 and RF2). This could be due to the formation of lipid complexes with starch and protein (Alam et al. 2015). The value of fat content observed in this study was slightly lower than the fat content (4.20%–12.74%) reported by Honi et al. (2018) in extruded orange‐fleshed sweet potato and bambara groundnut based snacks.

The carbohydrate content of all flour samples ranged from 65.43% to 72.08%. Samples EF2 had the highest, while RF1 contained the least carbohydrate content. Carbohydrate content in formulations containing maize was significantly higher than that in formulations without maize. This could be attributed to the fact that during the processing of maize, refined maize flour is found to contain higher levels of carbohydrates (Gwirtz and Garcia‐Casal 2014; Kamotho 2020). Extrusion cooking did not affect carbohydrate content significantly (*p* < 0.05); this is because carbohydrates are primarily composed of sugars, starches, and fiber, which are relatively stable compounds that are not significantly broken down by high temperatures during extrusion (Ito et al. 2015). Previous work has also reported a similar trend of no significant effect on carbohydrates by extrusion (Gui et al. 2012). However, extrusion can influence the availability and digestibility of carbohydrates through gelatinization of starches, hence making them more readily digestible (Omosebi et al. 2018).

The gross energy content of all flour samples ranged from 368.79 to 391.81 kcal. The highest being found in the control and the least in the formulation RF2. Formulations with maize exhibited lower gross energy compared to the formulations with maize. This could be attributed to the level of refinement during maize processing. The commonest means of processing maize results in low levels of fat and protein hence lower gross energy (Gallego‐Castillo et al. 2021; Kamotho et al. 2017). There was a significant difference (*p* < 0.05) in the gross energy content between raw and extruded composite flours. The gross energy content in extruded formulations was found to be higher than that in raw formulations. This could be because extrusion cooking breaks down complex molecules of starch and protein thereby making energy from these nutrients more readily available (Hejdysz et al. 2016).

### 3.6 Phytochemical Composition

#### 3.6.1 Total Antioxidant Capacity

The total antioxidant activity ranged from 137.45 mg AAE/100 g in control to 376.63 mg AAE/100 g in formulation RF1 (Table 7). The total antioxidant activity of the extruded formulations was significantly (*p* < 0.05) lower than that of raw formulations. This could be attributed to the degradation or loss of heat‐sensitive antioxidant compounds due to exposure to high temperatures during extrusion (Patil and Kaur 2018). Formulations containing maize had significantly (*p* < 0.05) lower total antioxidant capacity than formulations without maize. This could be attributed to the fact that processing maize, which involves the removal of bran and germ layers, leads to the loss of phytochemicals (Suri and Tanumihardjo 2016).

**TABLE 7:** Phytochemicals content of control and OFSP‐based composite flours.

Sample ID	Total antioxidants (mg AAE/100 g)	Flavonoids (mg CatE/100 g)	Total phenolics (mg GAE/100 g)	Tannins (mg CatE/100 g)	Phytates (mg/100 g)
COMM	137.45^d^ ± 9.58	225.47^b^ ± 6.98	167.80^d^ ± 3.90	14.81^e^ ± 1.55	1.61^d^ ± 0.00
RF1	376.63^a^ ± 3.52	575.38^a^ ± 5.15	537.37^a^ ± 3.25	97.51^a^ ± 6.99	1.70^b^ ± 0.02
RF2	327.70^ab^ ± 5.27	339.42^ab^ ± 4.99	444.30^ab^ ± 3.37	59.60^b^ ± 4.89	1.74^a^ ± 0.00
EF1	313.67^b^ ± 7.51	58.17^c^ ± 3.19	403.60^b^ ± 5.02	30.15^a^ ± 7.51	1.65^c^ ± 0.01
EF2	173.536^c^ ± 1.40	30.82^d^ ± 3.19	363.44^c^ ± 5.39	23.51^d^ ± 4.15	1.66^c^ ± 0.03

*Note:* Results are presented as mean ± SD. Means in the same columns with different superscripts are significantly (*p* < 0.05) different. Values with different superscripts in the same columns are significantly different, whereas values with the same superscript in the same columns are not significantly different.

Abbreviations: COMM: commercial composite flour (control); EF1, EF2: extruded composite formulations; RF1, RF2: raw composite formulations.

#### 3.6.2 Flavonoids Content

Flavonoid content varied between 30.82 and 575.38 mg CatE/100 for all samples. The highest flavonoid content was found in raw formulation RF1, while the least flavonoid content was recorded in extruded formulation EF2. The low values of flavonoid content in extruded formulations could be attributed to degradation due to the high temperatures involved (Patil and Kaur 2018). This is contrary to the report by Tiznado et al. (2013) who reported an increase in total flavonoids from 4.1% to 8.2% in extruded chickpeas. This could be due to differences in the raw materials composition (Bibi et al. 2022). However, it has been shown that extrusion processing can enhance the bioavailability of flavonoids by making them more accessible for absorption in the body (Patil and Kaur 2018).

#### 3.6.3 Total Phenolics Content

Total phenolics content ranged from 167.80 mg GAE/100 g in control to 537.37 mg GAE/100 g in raw formulation RF1. The total phenolics content of the extruded formulations was significantly (*p* < 0.05) lower than that of the raw formulations. Similar results were reported by (Wani and Kumar 2016). Phenolic compounds are heat liable and can break up when exposed to high temperatures, reducing their concentration (Patil and Kaur 2018). Therefore, losses in the total phenolics content of the formulations under extrusion are expected to occur due to the decomposition of the molecular structure of phenolic compounds, leading to the reduction in their chemical reactivity and extractability at high temperature conditions (Nayak et al. 2011).

#### 3.6.4 Tannins Content

Tannins ranged from 14.81 to 97.51 mg catE/100 g for all samples. The highest being recorded in RF1 and the least in EF2. Other studies (Omosebi et al. 2018; Wani and Kumar 2016) have reported a decrease in tannins content as a result of extrusion. The low values of tannins observed in extruded samples could be due to thermal breakdown due to extrusion cooking (Nikmaram et al., 2017). Formulations containing maize had significantly (*p* < 0.05) lower tannins content than formulations without maize. This could be attributed to the fact that refined maize flour, which was used for this study is expected to have a low content of phytochemicals including tannins (Suri and Tanumihardjo 2016).

#### 3.6.5 Phytates Content

The minimum (1.61 mg/100 g) and maximum (1.74 mg/100 g) content of phytate was obtained from control and raw formulation RF2, respectively. The total phytate content of the extruded formulations was significantly (*p* < 0.05) lower than that of the corresponding raw formulations. A similar trend was reported by Albarracin et al. (2015). Phytates are generally considered undesirable in foods since they form complexes with positively charged ions (Fe^2+^, Zn^2+^, Ca^2+^, Mg^2+^), charged groups of proteins, and can interact with starch molecules via hydrogen bonding with phosphate groups, making them unavailable for absorption and utilization by the body (Natabirwa et al. 2018; Neela and Fanta 2019). The observed low values of phytate content in extruded formulations are expected because extrusion cooking hydrolyzes phytates to release phosphate molecules and other components (Wani and Kumar 2016). The breakdown of phytate has several benefits in enhancing the bioavailability of minerals such as iron, zinc, and calcium that are bound to phytates, making them more accessible for absorption in the digestive tract.

### 3.7 Conclusion

Optimizing the formulation of orange‐fleshed sweet potato‐based composite flour supplemented with grain amaranth, biofortified beans, and maize flour was successfully done using RSM. Results show that nutrient enhanced OFSP‐based composite flour can be produced using the following optimal formulations with high desirability: RF1: OFSP (37.8%), maize flour (0.0%), bean flour (32.2%) and grain amaranth flour (30.0%) for those who are not highly dependent on maize, and RF2: OFSP (20.0%), maize flour (30.0%), bean flour (40.0%) and grain amaranth flour (10.0%) for raw composite flour. EF1: OFSP (46.6%), maize flour (0.0%), bean flour (36.9%) and grain amaranth flour (16.5%) and EF2: OFSP (49.0%), maize flour (5.9%), bean flour (35.1%) and grain amaranth flour (10.0%) for extruded composite flour. Special cubic models were found to adequately represent relations between data for the different variables (with *p* < 0.05). All the prediction models obtained using RSM were significant (*p* < 0.05) whereas lack of fit *p*‐values were non‐significant (with *p* > 0.05), which indicated the suitability of the models in predicting responses. Comparison of experimental values to those predicted for optimal formulations revealed no significant differences (*p* > 0.05). The study therefore resulted in optimal formulations that can be used for producing nutrient enhanced composite flour with OFSP, grain amaranth, biofortified bean, and maize flours as ingredients. The formulations could be a basis for the utilization of these locally available crops for the production of nutrient enhanced products that could be useful in addressing malnutrition in Uganda. Commercial application of these formulations would contribute to the alleviation of the challenge of poor market access by producers of the crops used in the formulations.

## Author Contributions

**Julius Byamukama:** conceptualization (equal), data curation (equal), investigation (equal), methodology (equal), software (equal), validation (equal), visualization (equal), writing – original draft (equal), writing – review and editing (equal). **Robert Mugabi:** conceptualization (equal), methodology (equal), software (equal), supervision (equal), validation (equal), visualization (equal). **John Muyonga:** conceptualization (equal), data curation (equal), funding acquisition (equal), investigation (equal), methodology (equal), project administration (equal), supervision (equal), writing – original draft (equal). **Dorothy Nakimbugwe:** conceptualization (equal), methodology (equal), supervision (equal), validation (equal), visualization (equal), writing – review and editing (equal).

## Conflicts of Interest

The authors declare no conflicts of interest.

## Data Availability

The data used to support the findings of this study are included in the article. Should further data or information be required, these are available upon reasonable request from the corresponding author.
